# Developmental Change of Yolk Microbiota and Its Role on Early Colonization of Intestinal Microbiota in Chicken Embryo

**DOI:** 10.3390/ani12010016

**Published:** 2021-12-22

**Authors:** Peng Ding, Huichao Liu, Yueyue Tong, Xi He, Xin Yin, Yulong Yin, Haihan Zhang, Zehe Song

**Affiliations:** 1 College of Animal Science and Technology, Hunan Agricultural University, Changsha 410128, China; shkdingpeng@163.com (P.D.); liuhc1@126.com (H.L.); ty9824@126.com (Y.T.); hexi111@126.com (X.H.); 18374874243@163.com (X.Y.); yinyulong@isa.ac.cn (Y.Y.); 2 Ministry of Education Engineering Research Center of Feed Safety and Efficient Use, Changsha 410128, China; 3 Hunan Engineering Research Center of Poultry Production Safety, Changsha 410128, China; 4 Hunan Co-Innovation Center of Animal Production Safety, Changsha 410128, China

**Keywords:** yolk, microbiota, intestine, embryo, colonization

## Abstract

**Simple Summary:**

For nearly a century, genetic selection and improved management have led to significant progress in poultry’s productive traits. The embryonic development of commercial broiler chickens, which has become a larger part of the production cycle of broiler chickens (approximately 33%), has attracted more and more attention. It has been shown in previous studies that microbes played an important role in animal growth and development. The yolk is an important source of nutrients for chicken embryonic development. At late embryonic development, the remaining yolk will enter the embryo intestine directly through the yolk stem and may contribute to the formation of intestinal microbiota. Therefore, we investigated the developmental change of yolk microbiota and its role on early colonization of intestinal microbiota in the chicken embryo. The results showed that the relative abundance of yolk microbiota was barely changed during embryogenesis. According to the KEGG analysis, the yolk microbiota were functionally related to amino acid, carbohydrate, and lipid metabolisms during chicken embryogenesis. The yolk microbiota influenced the embryonic intestinal microbiota by increasing the colonization of Proteobacteria, Firmicutes, and Bacteroidetes in the intestine.

**Abstract:**

Although the fertilized eggs were found to contain microbes in early studies, the detailed composition of yolk microbiota and its influence on embryo intestinal microbiota have not been satisfactorily examined yet. In this study, the yolk microbiota was explored by using 16s rRNA sequencing at different developmental stages of the broiler embryo. The results showed that the relative abundance of yolk microbiota was barely changed during embryogenesis. According to the KEGG analysis, the yolk microbiota were functionally related to amino acid, carbohydrate, and lipid metabolisms during chicken embryogenesis. The yolk microbiota influences the embryonic intestinal microbiota through increasing the colonization of Proteobacteria, Firmicutes, and Bacteroidetes in the intestine, particularly. The intestinal microbes of neonatal chicks showed higher proportions of *Faecalibacterium*, *Blautia*, *Coprococcus*, *Dorea*, and *Roseburia* compared to the embryonic intestinal microbiota. Our findings might give a better understanding of the composition and developmental change of yolk microbiota and its roles in shaping the intestinal microbiota.

## 1. Introduction

The intestinal microbiota transferred from the mother to the embryo, which is mainly obtained from the bacteria resident in the maternal reproductive tract, has been shown to have a significant influence on the colonization of microbiota in the gut of human newborns [1,2,3]. However, for avian species, whose embryo develops separately from the maternal surrogate, the embryonic microbiota are also possibly affected by the external environment and internal nutrient changes.

During incubation, embryonic growth and development are dependent on nutrients deposited in the egg. The yolk is an important source of nutrients for chicken embryonic development and contains lipids and proteins derived from the maternal liver [4]. The yolk content can be transferred to the embryo in 2 ways: either through the highly vascularized yolk sac membrane or directly in the intestine via the yolk stalk [5,6]. During early embryonic development, the yolk nutrients are primarily absorbed and metabolized into small molecules such as lipoprotein, carbohydrate, amino acid, or fatty acid through the enzymatic digestion of the yolk sac [7,8]. At the late embryonic development (approximately embryonic day 17–21), even within 6 days after chickens hatched, the yolk sac is atrophied gradually. Meanwhile, the is transported through the yolk stalk into the embryo intestines. The direct entry of yolk into the embryo intestine promotes the growth and development of the intestine, and the establishment of the early microbiome of the chicken intestine [9].

Using modern sequencing technology, the composition and presence of microbiota in the neonatal chicken gut were found to be determined by either the inheritance of the microbiome of the maternal oviduct or the acquisition of bacteria from the external environment on hatch [10,11,12,13]. Previous studies on the intestinal microbiota of chicken embryos are relatively rare. However, the current studies have shown that the growth performance and post-hatch health of chicken embryos were improved by in ovo feeding of probiotics or prebiotics [11,14,15,16]. Such beneficial effects are achieved by altering the microbiota within the eggs. A possible reason for the intestinal microbiota colonization of newly hatched chickens could be the microbial translocation from the yolk to the intestine. However, the influence of yolk microbes on shaping the embryo intestinal microbiota is still unknown. Additionally, the profile of bacteria in the egg yolk at different embryonic stages might be dynamically changed due to the nutrients in the yolk contents being continuously utilized. Thus, in this study, we investigated the developmental change of microbiota in the fertilized egg yolk and tried to explore the potential importance of yolk microbes in forming the microbiome in the chicken embryonic intestine.

## 2. Material and Methods

### 2.1. Animals and Sample Preparation

A total of 200 fertilized eggs of Ross 308 commercial broilers were purchased from Shuncheng broiler breeder farm (Ningxiang, China). All the eggs were collected from the 35-week old flocks. The animal procedures in this study were approved by the institutional animal care and use committee of Hunan Agricultural University. Eggs were subjected to a standardized cleaning and disinfection procedure by steaming with formaldehyde and potassium permanganate solution (2:1) for 30 min before incubating at 37.8 °C with 60–80% humidity. The incubators were sterilized by ultraviolet light sanitization before use. The ventilation system was connected to the common environmental air supply without any sterilizing treatments. Randomly select 6 fertilized eggs at each time point to collect samples. To investigate the microbiota in egg yolk, at each sampling day, the eggshell was gently peeled off using a sterile tweezer to expose the embryo. Yolk samples (5 mL) were collected by using a syringe to puncture through the yolk sac membrane and homogenize them before freezing the samples in liquid nitrogen. Dissect the abdomen of chicken embryos with a scalpel and carefully remove the whole gut on a 4 °C sanitized working bench, subsequently, stored at −80 °C after snap freezing in the liquid nitrogen.

### 2.2. DNA Extraction and 16s rRNA Sequencing

Yolk samples (5 mL) were thawed on ice, then put in 10 mL sterile 1X PBS buffer to remove the excessive yolk fat and centrifuged at 16,000 rpm for 5 min. The total DNA was extracted from embryonic intestinal samples after homogenization. The DNA from the samples were extracted by using TIANGEN DNA Stool Mini Kit (TIANGEN, cat#DP328, Beijing, China) and following the manufacturer’s instructions. The concentration and quality of isolated DNA were assessed by using a NanoDrop spectrophotometer (Thermo Fisher Scientific, Waltham, MA, USA). Amplicons of V4, V5 hypervariable regions of 16s rRNA were amplified by using the sample-specific sequence barcoded fusion primers (forward 5′-GTGCCAGCMGCCGCGGTAA-3′ and reverse 5′-CCGTCAATTCMTTTRAGTTT-3′). The thermal cycle consisted of an initial denaturation at 98 °C for 2 min, followed by 25 cycles including denaturation at 98 °C for 15 s, annealing at 55 °C for 30 s, extension at 72 °C for 30 s, and a final extension at 72 °C for 5 min. PCR amplicons were purified with Agencourt AMPure Beads (Beckman Coulter, Indianapolis, IN, USA) and quantified using the PicoGreen dsDNA Assay Kit (Invitrogen, Carlsbad, CA, USA). 16s rRNA sequencing was performed using Illumina Novaseq_PE250 (Illumina) sequencing platform, sequencing services were provided by Personal Biotechnology Co., Ltd. Shanghai, China. The data were analyzed by using the free online platform Personalbio GenesCloud, https://www.genescloud.cn (accessed on 29 October 2021).

### 2.3. Sequence Analysis

In total, 4930714 high-quality reads were generated for further analyses. The Quantitative Insights Into Microbial Ecology (QIIME2, 2019.4) pipeline was used to process the sequencing data [17]. In short, raw sequencing reads that exactly match the barcode are assigned to their respective samples and are determined to be valid sequences. Low-quality sequences are filtered by the following criteria [18,19]: sequences that had a length of <150 bp, sequences that had average Phred scores of <20, sequences that contained ambiguous bases, and sequences that contained single nucleotide repeats of >8 bp. Paired-end reads were assembled using FLASH [20]. After chimera detection, the remaining high-quality sequences were clustered by UCLUST into operational taxonomic units (OTUs) with 97% sequence identity [21]. Select a representative sequence from each OTU using the default parameters. Search the set of representative sequences by BLAST with Greengenes database for OTU classification [22] using the best hit [23].

### 2.4. Bioinformatics and Statistical Analysis

Sequence data analyses were mainly performed using QIIME and R packages (v3.2.0). OTU-level alpha diversity indices, such as Chao1 richness estimator, ACE metric (Abundance-based Coverage Estimator), Shannon diversity index, and Simpson index, were calculated using the OTU table in QIIME. OTU-level ranked abundance curves were generated to compare the richness and evenness of OTUs among samples. Beta diversity analysis of structural changes in microbial communities of different samples using non-metric multidimensional scaling (NMDS) and unweighted pair group method (UPGMA) hierarchical clustering [24]. Principal component analysis (PCA) was also conducted based on the genus-level compositional profiles [24]. The significance of differentiation of microbiota structure among groups were assessed by PERMANOVA (Permutational multivariate analysis of variance) [25] and ANOSIM (Analysis of similarities) [26,27] using the R package “vegan”. The taxonomy compositions and abundances were visualized using MEGAN [28] and GraPhlAn [29]. Use the R package “VennDiagram” to generate a Venn diagram to visualize shared and unique OTUs between samples or groups based on the occurrence of OTUs between samples/groups, regardless of their relative abundance [30]. Taxa abundances at the phylum, class, order, family, and genus levels were compared by Metastats statistics for each sample or group [31] and visualized as violin plots. LEfSe (Linear discriminant analysis effect size) was used to detect taxa of different abundance in different groups, using the default parameters [32]. PLS-DA (Partial least squares discriminant analysis) was also introduced as a supervised model to reveal the microbiota variation among groups, using the “plsda” function in the R package “mixOmics” [33]. Microbial functions were predicted by PICRUSt (Phylogenetic investigation of communities by reconstruction of unobserved states), based on high-quality sequences [34]. The proportion of intestine sample microbiota is estimated to originate from yolk sources using bacterial source-tracking [35,36].

## 3. Results

### 3.1. Composition of Microbiota in Yolk and Gut of Chicken Embryo

**Figure 1:**
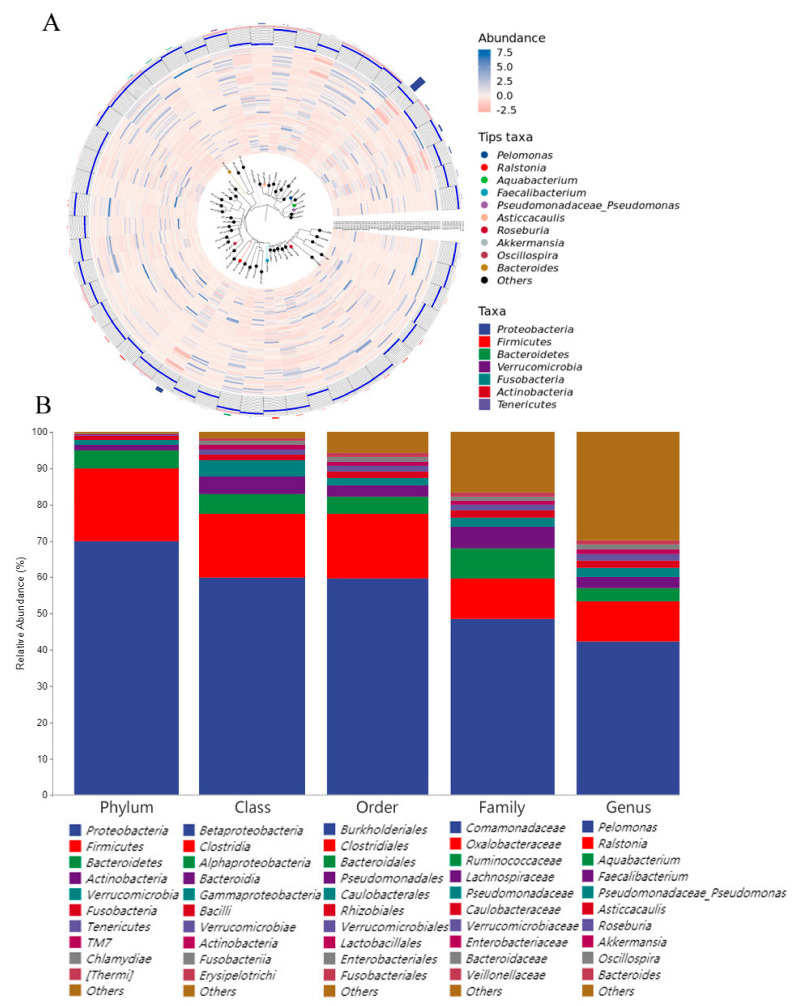
Composition of yolk microbiota and intestinal microbiota in the chicken embryo. (**A**) Phylogenetic tree constructed from the taxa. According to the selected taxonomic level, OTU representative sequences (tips in the figure) and their connected branches are colored, and specific species can be selected for marking according to the needs. (**B**) The abundant microbes at each level (genus, family, order, class, and phylum).

Thirty-six samples were collected in the whole experiment, including yolk and gut samples. 6 yolk samples were collected at each sampling day (E07, E11, E15, and E19) and 6 gut samples from chicken embryos were collected at E19 and the day of hatch (DOH), respectively. The total number of operational taxonomical units (OTU) identified in the yolk and intestine was 3836. The microbes with the most abundance detected at the phylum level were Proteobacteria, Firmicutes, Bacteroidetes, Verrucomicrobia, Fusobacteria, Actinobacteria, and Tenericutes; *Pelomonas*, *Ralstonia*, *Aquabacterium*, *Faecalibacterium*, *Pseudomonadaceae*, *Asticcacausia*, *Roseburia*, *Akkermansia*, *Oscillospira*, and *Bacteroides* at the genus level (Figure 1A). The detailed classification of specific taxon groups was investigated, and the results showed that 17 phyla, 33 classes, 51 orders, 88 families, and 125 genera existed in the yolk and embryo gut. The phylum clustered with the highest abundance was Proteobacteria (69.81%), followed by Firmicutes (20.14%), Bacteroidetes (4.90%), Actinobacteria (1.54%), and Verrucomicrobia (1.41%). According to the assignment of OTUs at the genus level, *Pelomonas* (42.30%) and *Ralstonia* (11.08%) shared with the most abundant OTUs, and they belonged to Proteobacteria and Firmicutes which were the top two abundant bacteria of phylum in yolk and embryo gut. Additionally, *Aquabacterium* (3.53%), *Faecalibacterium* (3.22%), *Pseudomonas* (2.41%), and *Asticcacaulis* (1.93%) were detected at the genus level (Figure 1B). The above results confirmed that microorganisms existed in the yolk and intestine of chicken embryos.

### 3.2. Change of Yolk Microbiota at Different Developmental Stages

**Figure 2:**
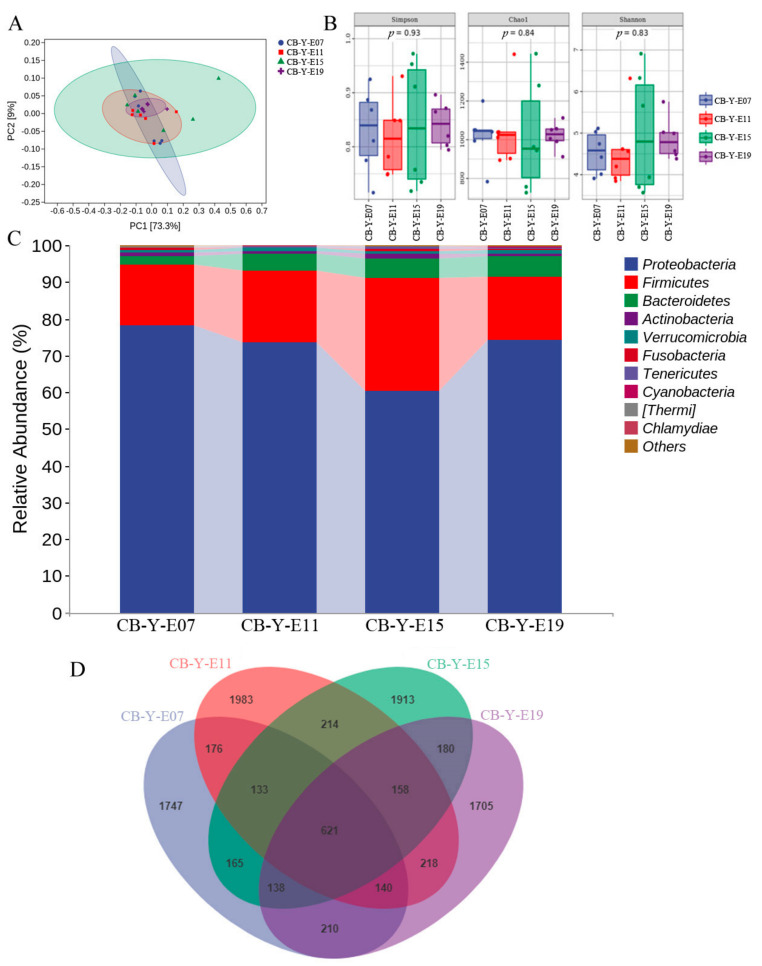

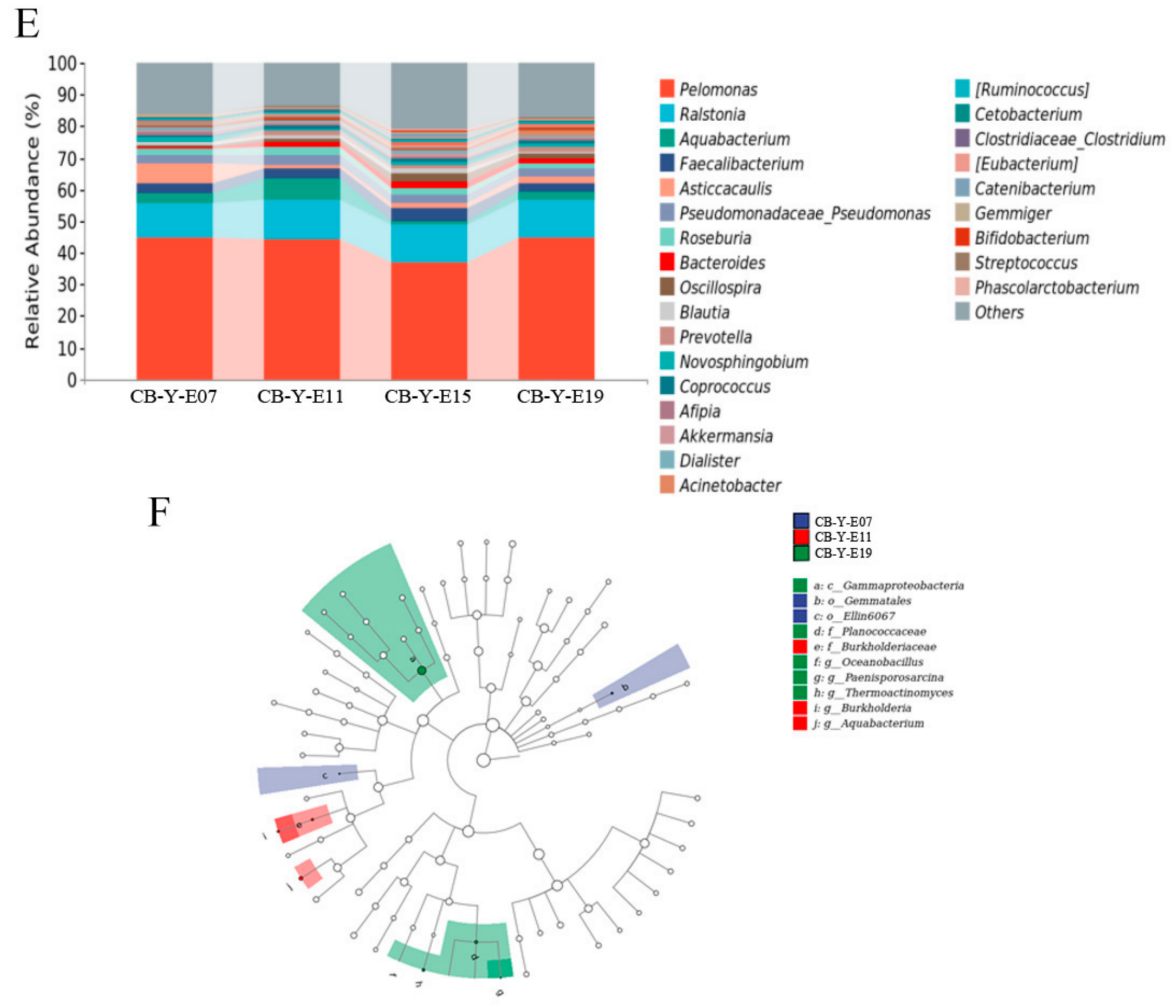
Embryonic yolk microbial difference at different developmental stages. CB in the picture represents commercial broilers and Y represents yolk. (**A**) PCA: Each point represents a sample, and points of different colors indicate different groups. (**B**) Each panel corresponds to an alpha diversity index, which is identified in the gray area at the top. In each panel, the abscissa is the group label, and the ordinate is the value of the corresponding alpha diversity index. (**C**) The relative abundance of yolk microbiota in the chicken embryo at different days. (**D**) The Venn diagram shows the core microbes shared at different stages of development. (**E**) The relative abundance of yolk microbiota in the chicken embryo at different days (at the genus level). (**F**) Taxonomic cladogram generated from LEfSe showing significant difference in the microbiota profile of 4 stages of development.

The PCA results showed that the clustering together of yolk samples, which explain the yolk microbial composition at each developmental stage had certain similarities (Figure 2A). Moreover, the variability of the microbial composition of individuals at E15 displayed more discrete compared with other earlier or later stages. It could be conjectured that the yolk microbiome is relatively stable during the whole embryogenesis, but more fluctuated at E15, probably due to the fast metabolic change of yolk nutrients at this stage. Figure 2B showed different diversity indexes estimating microbial richness or diversity, including Simpson index (*p* = 0.93), Chao1 index (*p* = 0.84), and Shannon index (*p* = 0.83). Then we compared the relative abundance of yolk microorganisms at different stages of chicken embryonic development. The results showed that Proteobacteria, Firmicutes, and Bacteroidetes were dominant at the phyla level at all 4 different stages, followed by Actinobacteria, and Verrucomicrobia. Additionally, the percentage of Firmicutes (30.81%) in the yolk reached the peak at E15 compared with E07 (16.49%) and E11 (19.35%) and further dropped at the E19 (17.26%), however, no significant difference was found in statistical analysis (*p* = 0.29) (Figure 2C). The proportion of Proteobacteria at E07 (78.33%), E11 (73.65%), and E15 (60.35%) showed a decreasing trend but increased at E19 (74.09%) in the egg yolk (*p* = 0.32). We further investigated the change of microbiome in the yolk, which were annotated from the OTUs identified at all different stages simultaneously. The Venn diagram showed 621 common OTUs shared between E07, E11, E15, and E19 (Figure 2D). The dominant microbes obtained from these 621 common OTUs were *Pelomonas* and *Ralstonia*. Additionally, compared across different embryonic days, *Asticcacaulis* and *Aquabacterium* were the bacteria highly enriched at E07 and E11, respectively (Figure 2E). Finally, the landmark bacteria at each developmental stage which were explored by LEfSe indicated that the relative levels of *Thermoactinomyces*, *Paenisporosarcina*, and *Oceanobacillus* were enriched in the yolk at E19, while the levels of *Burkholderia* and *Aquabacterium* were increased at E11 (Figure 2F).

### 3.3. Functional Profiles of Yolk Microbial Community during Embryogenesis

**Figure 3:**
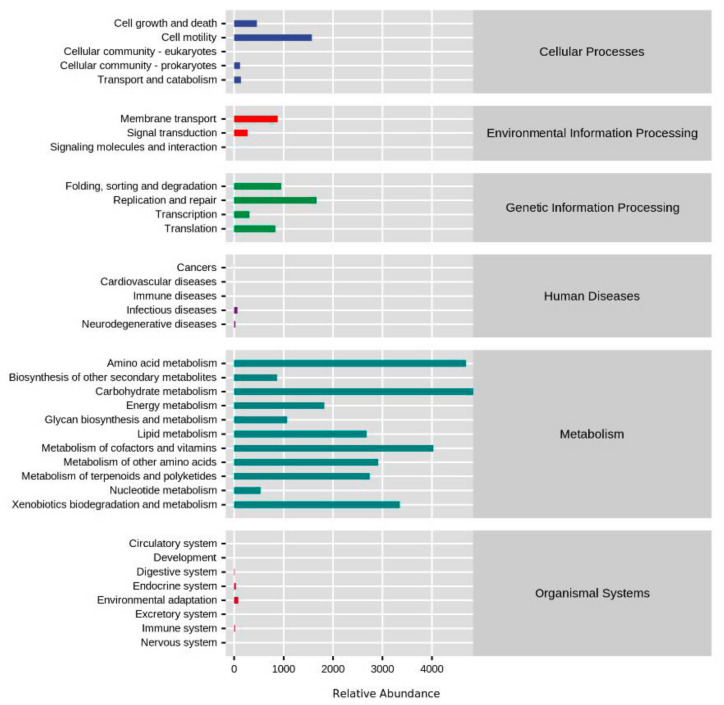
Functional profiles of the microbial community in the yolk. The abscissa is the abundance of the functional pathway, the ordinate is the functional classification of KEGG in the second level, and the rightmost is the first level of the pathway. Here is the average abundance of all selected samples or the count of all selected samples.

To understand the role of yolk microbiota particularly for the development of the chicken embryo, we analyzed metagenomic functions of the bacteria detected in the egg yolk (Figure 3). According to the second level of KEGG functional profiles, the results showed that yolk microbiota were mainly associated with the functions of amino acids, carbohydrates, lipids, cofactors, and vitamins metabolisms. Additionally, some yolk bacteria may participate in the activities related to nucleotide metabolism and genetic information processing perhaps due to the rapid cell growth and proliferation during embryonic development.

### 3.4. Comparison of the Microbiota between Yolk and Intestine at E19

**Figure 4:**
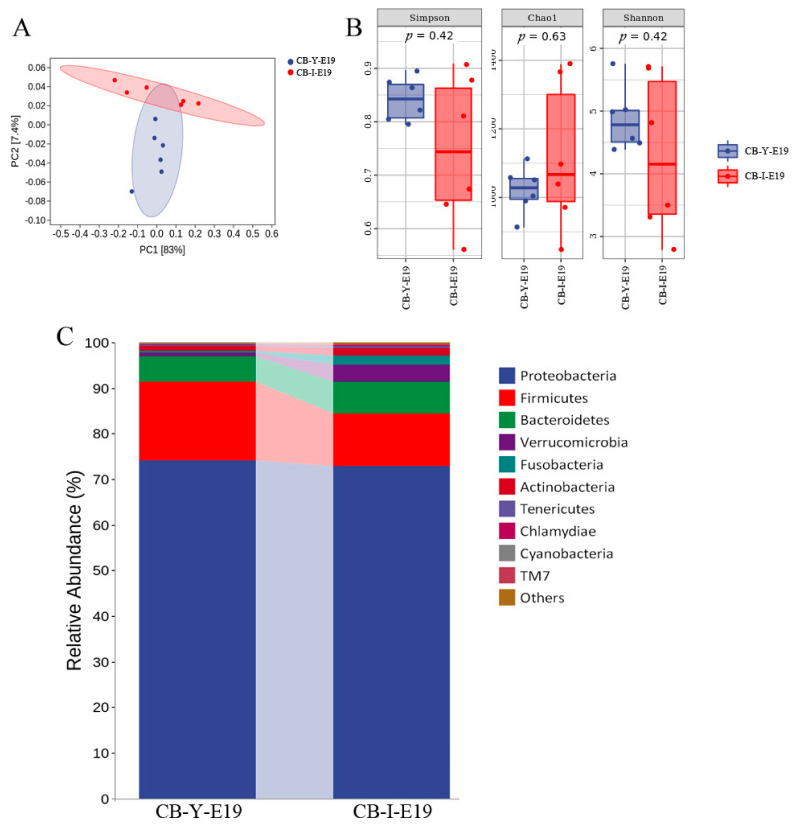

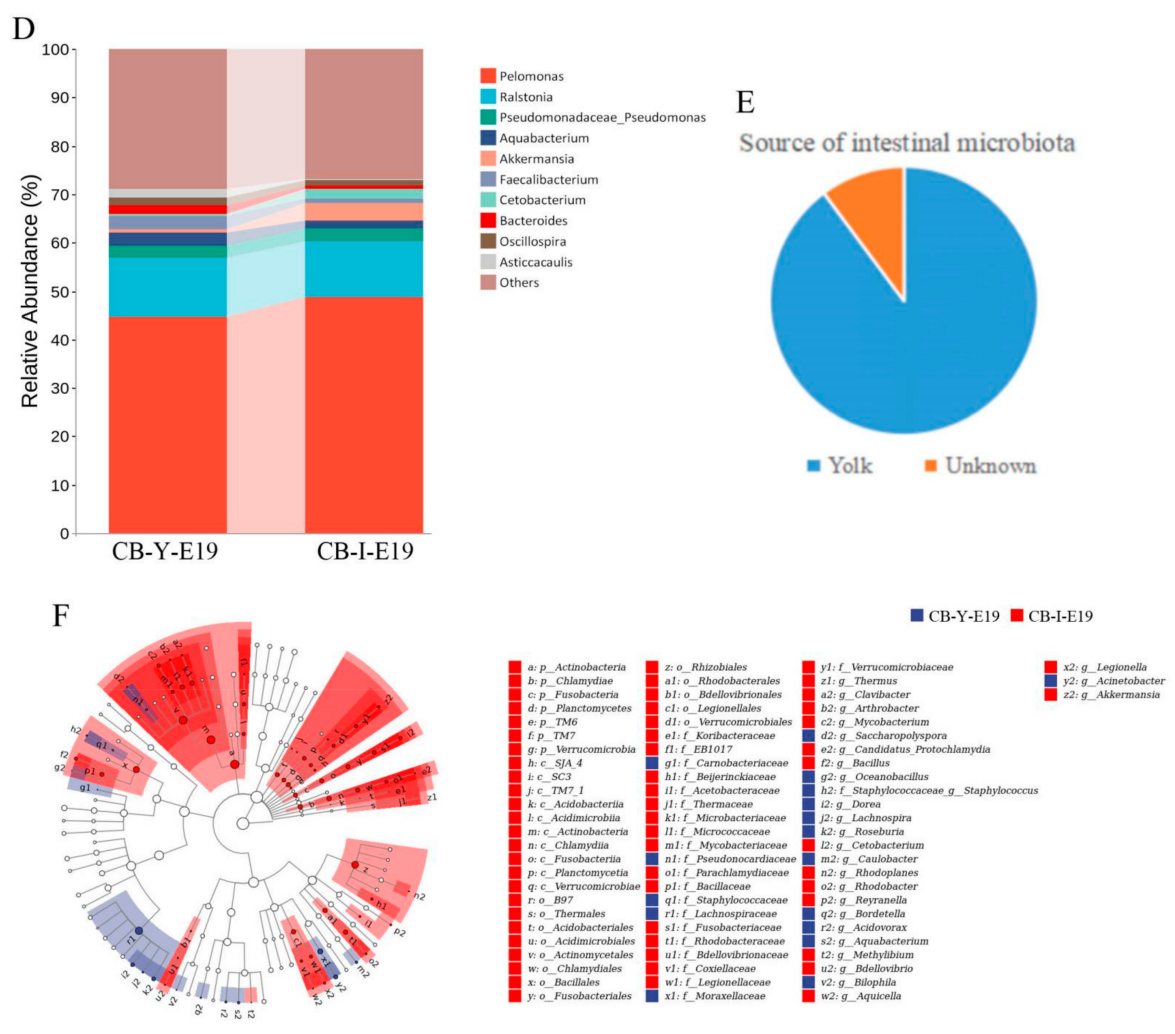
Comparison of embryonic microbiota between yolk and intestine and Source Tracker proportion estimates for a subset of sink samples. (**A**) PCA: Each point represents a sample, and points of different colors indicate different groups. (**B**) Each panel corresponds to an alpha diversity index, which is identified in the gray area at the top. In each panel, the abscissa is the group label, and the ordinate is the value of the corresponding alpha diversity index. (**C**) The relative abundance of yolk microbiota and intestinal microbiota at E19 (at the phylum level). (**D**) The relative abundance of yolk microbiota and intestinal microbiota at E19 (at the genus level). (**E**) Intestinal microbiota source of chicken embryos, using bacterial source-tracking. (**F**) Taxonomic cladogram generated from LEfSe showing significant difference in the microbiota profile of yolk microbiota and intestinal microbiota at E19. CB in the picture represents commercial broilers, Y represents yolk, and I represents intestine.

According to PCA, we found that the clustering of yolk microbiota and intestinal microbiota were different (Figure 4A). However, the alpha analysis showed that there was no significant difference between yolk and intestinal microbes, which was proved by the Simpson index (*p* = 0.42), Chao1 index (*p* = 0.63), and Shannon index (*p* = 0.42) (Figure 4B). Then we compared the relative abundance of microbiota between yolk and intestine at E19. The results showed that Proteobacteria, Firmicutes, and Bacteroidetes were dominant bacteria at the phyla level in the yolk and intestine, followed by Verrucomicrobia. The percentage of Firmicutes in the yolk was 17.26% and decreased to 11.56% in the intestine at E19 (*p* = 0.20). Additionally, the percentage of Verrucomicrobia significantly increased to 3.62% in the intestine compared to the yolk (0.77%) (*p* = 0.04) (Figure 4C). At the genus level, *Pelomonas* (44.67, 48.65%) and *Ralstonia* (12.11, 11.72%) were dominant bacteria in the yolk and intestine. It is worth noting that the relative abundance of *Akkermansia* in the intestine (0.74%) is significantly higher than that in the yolk (3.52%) (*p* = 0.04) (Figure 4D). Furthermore, LEfSe results showed the landmark microbes in the intestine were more abundant than those in the yolk at the genus level (Figure 4F). Notably, the intestinal bacteria appeared more likely to be Actinobacteria, Chlamydiae, Fusobacteria, Planctomycetes, TM6, TM7, and Verrucomicrobia at phylum level compared to the yolk microbes. The microbial composition and relative abundance of the dominant bacteria, Proteobacteria and Firmicutes at phylum level in the yolk and intestine were similar to each other, but some bacteria showed distinct at the genus level. This might indicate that the majority of the yolk microbiota was transferred to the intestine, but probably due to the physiological difference, the dominant bacteria at the genus level were shifted and adapted to the environment in the intestine without changing the phylum composition. According to source-tracking, we found that approximately 89.83% of the intestinal microbiota of chicken embryos was originated from the yolk (Figure 4E), which further confirmed our conjecture.

### 3.5. Transition of Intestinal Microbiota from Late Embryonic to Early Posthatch Stage

**Figure 5:**
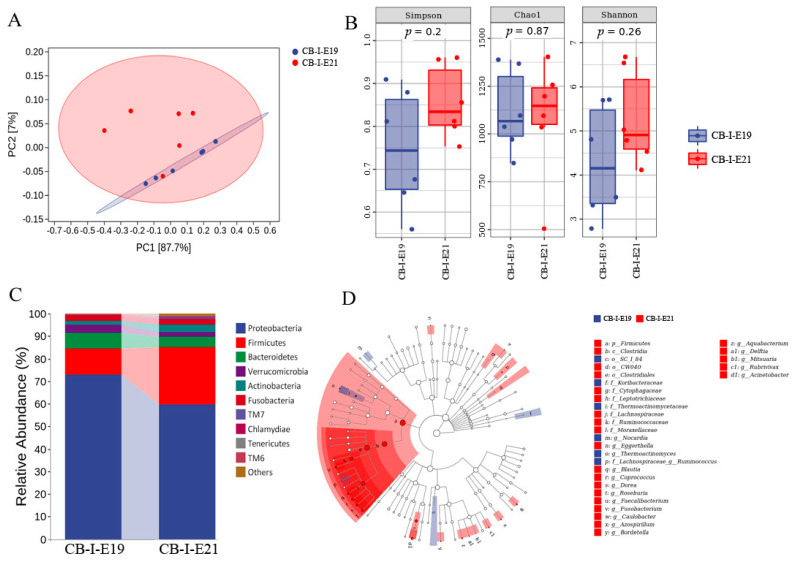
Early changes of intestinal microbiota in chicken embryos. (**A**) PCA: Each point represents a sample, and points of different colors indicate different groups. (**B**) Each panel corresponds to an alpha diversity index, which is identified in the gray area at the top. In each panel, the abscissa is the group label, and the ordinate is the value of the corresponding alpha diversity index. (**C**) The relative abundance of intestinal microbiota at E19 and E21 (at the phylum level). (**D**) Taxonomic cladogram generated from LEfSe showing significant difference in the microbiota profile of intestinal microbiota at E19 and E21. CB in the picture represents commercial broilers and I represent intestine.

The early colonization of intestinal microbiota for post-hatch chickens is related to the feed and environmental microbes. However, the formation of the hatchling’s microbiota should be retrieved around the late embryonic stage when the chicken embryo pecks off the eggshell and begins to breathe [10]. Thus, we compared the intestinal microbiota at E19 and E21. According to PCA, we found that the composition of intestinal microbes at E21 fluctuated than that at E19, which were clustered more closely (Figure 5A). The alpha-diversity analysis, including Simpson index (*p* = 0.20), Chao1 index (*p* = 0.87), and Shannon index (*p* = 0.26), indicated that there was no significant difference between E19 and E21 of intestinal microbes (Figure 5B). The comparison of intestinal microbial relative abundance between E19 and E21 demonstrated that the percentage of Proteobacteria was declined from E19 (72.78%) to E21 (59.66%) (*p* = 0.17). Meanwhile, the percentage of Firmicutes in the intestine was significantly elevated from E19 (11.56%) to E21 (25.37%) (*p* = 0.04) (Figure 5C). Further LEfSe analysis verified that the bacteria of Firmicutes were enriched in the hatchling’s intestine, and the corresponding dominant microbes at genus level were *Blautia*, *Coprococcus*, *Dorea*, *Roseburia*, *Faecalibacterium*, particularly (Figure 5D).

## 4. Discussion

At present, most of the studies about chicken intestinal microbiota are focused on the post-hatch period, and only a few reports were carried out to look at the changes of microbiota during embryogenesis [10,13,37]. The main source of nutrients for chicken embryo development is the yolk, which provides more than 90% of the nutrients and is dynamically metabolized to supply essential elements for embryonic organ growth and tissue formation. Our earlier research found that the yolk was probably not sterile due to the identification of microbial metabolites in the yolk [38]. However, the change of yolk microbiota during the chicken embryonic development and the effects on early colonization of the intestinal microbiota were not clear yet. This study was designed to bridge this gap by examining the dynamic change of yolk microbiota and intestinal microbiota in broiler chickens at different embryonic stages.

Our results showed that the yolk microbiota fluctuated little during the chicken embryogenesis. However, we observed an increase of Firmicutes relative abundance in the yolk microbial population as the embryos developed from E7 to E15. Interestingly, the ratio of Firmicutes to Bacteroidetes was gradually increased from E7 to E15 but decreased from E15 to E19, and the previous publications indicated that Firmicutes to Bacteriodetes ratio might be associated with the nutrient absorption and weight gain [39,40,41]. The utilization of egg yolk nutrients was either to support the growth of the yolk sac membrane or to generate developmental materials for the embryo. With the evidence that the weight of the yolk sac membrane reached the top at E15 during the embryogenesis [42], the ratio of Firmicutes to Bacteroidetes in egg yolk microbiota might be a reason to influence the yolk sac membrane growth. The major nutrients in the egg yolk are lipids, which account for 31–33% and most of them are in the form of very-low-density lipoproteins. We summarized the OTUs that shared with all the embryonic stages (Figure 2D) and assumed them as microbes that might be associated with the fundamental metabolic functions such as lipids metabolism or amino acids metabolism. Then, the relative abundances of the bacteria that were annotated with the shared OTUs were calculated (Figure 2E). The results indicated that *Asticcacaulis* was the microbe with the highest relative abundance at E07, and the peak relative abundance of *Aquabacterium* was at E11, *Pelomonas* was the bacteria with the lowest relative abundance at E15. In contrast to *Aquabacterium* and *Pelomonas*, which were potentially pathogenic bacteria, *Ralstonia* and *Faecalibacterium* were the dominant and beneficial bacteria. The relative abundance of *Ralstonia* and *Faecalibacterium* in the yolk displayed almost constant throughout the embryogenesis. Previous reports showed that the fluctuation of *Faecalibacterium* or *Ralstonia* in the microbiota of patients was associated with metabolic disorder and inflammation, such as nonalcoholic steatohepatitis or obesity [43,44]. The metabolic rates of chicken embryo liver and yolk sac were really active due to the vast consumption of lipids for energy supply and embryo growth during embryogenesis [6,45,46]. Thus, the constant abundance of *Faecalibacterium* and *Ralstonia* from E07 to E19 in the yolk was probably related to the maintenance of normal lipids metabolic reactions during this stage in the egg. It is worth mentioning that the rapid metabolism of lipids in the organism is usually accompanied by oxidative stress and cell damage, which do not seem to be present in the rapid development of chicken embryos. Whether the yolk microbiota plays an important role in this process is an interesting and worthwhile question for further research.

To understand the potential functions of the yolk microbiota, we categorized them into different functional pathways (Figure 3). The results indicated that the most of yolk microbiota were associated with the functions of amino acids metabolism, carbohydrates metabolism, vitamins and cofactors metabolism, and lipids metabolism. Yolk nutrients were primarily served as the fuels for embryo development and growth, and have to be absorbed by the yolk sac membrane initially. The expression of yolk sac nutrient transporters showed that amino acid, glucose, and lipid absorption rapidly occurred during chicken embryonic development [7,8,47]. Thus, the yolk microbiota may play a role in metabolizing yolk nutrients for embryo development.

The organogenesis of the chicken embryo is fully developed already around the last two days of the incubation when the yolk sac is internalized in the chicken abdomen. The early formation of the chicken intestine also begins at the end of embryogenesis [48,49], and it is reasonable to infer that the microbial colonization might be initiated at the late embryonic stage. One thing which is worthy to be noticed is that the yolk residues are squeezed into the intestine through the yolk stalk and determine the early intestinal microbiota probably close to the end of the incubatory stage. Thus, we compared the yolk microbiota and the intestinal microbiota at E19 to investigate the potential influence of yolk microbiota on early colonization of the intestinal microbiota. The results showed that the relative abundance of the dominated microbes in the yolk and intestine were similar (Figure 4C). The resemblance of yolk microbiota and intestinal microbiota might support our assumption that the yolk microbiota contributes to the formation of embryonic intestinal microbiota. However, due to the physiological and functional differences between the yolk sac and the intestinal epithelium, some discrepancies might be observed. Therefore, the representative bacteria were detected in the yolk and intestine, respectively (Figure 4F). Prominently, Actinobacteria showed high enrichment in the chicken intestine, which was consistent with the previous report that Actinobacteria was one of the conserved bacteria in the chicken embryo intestine [10]. Thus, we might assume that the yolk microbiota made a great contribution to the early colonization of the dominated intestinal bacteria including Proteobacteria, Firmicutes, Bacteroidetes, and Verrucomicrobia. The bacterial source-tracking analysis also proved our results.

The early colonization of the chicken embryo intestine also depends on exposure to the external environment. So, we compared the intestinal microbiota at E19 and E21 (the day of hatch) to understand the potential effects of environmental exposure on the formation of their intestinal microbiome. Strikingly, when the chickens were hatched, the population of Firmicutes was increased and the Proteobacteria proportion decreased. At the genus level, *Blautia*, *Coprococcus*, *Dorea*, *Roseburia*, and *Faecalibacterium* were the beneficial bacteria highly enriched in the neonatal chicks. Due to these dominated bacteria were functionally related to healthy body condition and host invulnerability [50,51,52,53,54], the newly-hatched chicks may need to get these bacteria involved in the microbiome for improving the viability of the early post-hatch stage.

## 5. Conclusions

In conclusion, our results found that the yolk microbiota did not change significantly during the chicken embryonic development, and the yolk microbiota were primarily associated with the function of amino acids, carbohydrates, and lipids metabolisms. The yolk microbiome might contribute to the formation of chicken embryonic intestinal microbiota, particularly relying on the colonization of Proteobacteria, Firmicutes, and Bacteroidetes. The intestinal microbiota of newly-hatched chickens differs from the embryonic intestinal microbiome, mainly in elevating the percentage of beneficial bacteria including *Faecalibacterium*, *Blautia*, *Coprococcus*, *Dorea*, and *Roseburia*. Our findings might provide a novel insight into understanding the composition of the yolk microbiota and its function in shaping the intestinal microbiota of chicken embryos.

## Data Availability

The datasets generated for this study can be found in the NCBI—SRR14887295.

## References

[1] Doyle R.M., Alber D.G., Jones H.E., Harris K., Fitzgerald F., Peebles D., Klein N. (2014). Term and preterm labour are associated with distinct microbial community structures in placental membranes which are independent of mode of delivery. Placenta.

[2] Walther-Antonio M.R., Chen J., Multinu F., Hokenstad A., Distad T.J., Cheek E.H., Keeney G.L., Creedon D.J., Nelson H., Mariani A. (2016). Potential contribution of the uterine microbiome in the development of endometrial cancer. Genome Med..

[3] Yang I., Corwin E.J., Brennan P.A., Jordan S., Murphy J.R., Dunlop A. (2016). The Infant Microbiome: Implications for Infant Health and Neurocognitive Development. Nurs. Res..

[4] Vieira S.L. (2007). Chicken embryo utilization of egg micronutrients. Braz. J. Poult. Sci..

[5] Bauer R., Plieschnig J.A., Finkes T., Riegler B., Hermann M., Schneider W.J. (2013). The developing chicken yolk sac acquires nutrient transport competence by an orchestrated differentiation process of its endodermal epithelial cells. J. Biol. Chem..

[6] Yadgary L., Wong E.A., Uni Z. (2014). Temporal transcriptome analysis of the chicken embryo yolk sac. BMC Genom..

[7] Speier J.S., Yadgary L., Uni Z., Wong E.A. (2012). Gene expression of nutrient transporters and digestive enzymes in the yolk sac membrane and small intestine of the developing embryonic chick. Poult. Sci..

[8] Zhang H., Wong E.A. (2017). Spatial transcriptional profile of PepT1 mRNA in the yolk sac and small intestine in broiler chickens. Poult. Sci..

[9] Van der Wagt I., de Jong I.C., Mitchell M.A., Molenaar R., van den Brand H. (2020). A review on yolk sac utilization in poultry. Poult. Sci..

[10] Akinyemi F.T., Ding J., Zhou H., Xu K., He C., Han C., Zheng Y., Luo H., Yang K., Gu C. (2020). Dynamic distribution of gut microbiota during embryonic development in chicken. Poult. Sci..

[11] Cisek A.A., Binek M. (2014). Chicken intestinal microbiota function with a special emphasis on the role of probiotic bacteria. Pol. J. Vet. Sci..

[12] Lee S.J., Cho S., La T.M., Lee H.J., Lee J.B., Park S.Y., Song C.S., Choi I.S., Lee S.W. (2020). Comparison of microbiota in the cloaca, colon, and magnum of layer chicken. PLoS ONE.

[13] Lee S., La T.M., Lee H.J., Choi I.S., Song C.S., Park S.Y., Lee J.B., Lee S.W. (2019). Characterization of microbial communities in the chicken oviduct and the origin of chicken embryo gut microbiota. Sci. Rep..

[14] Cuperus T., Kraaij M.D., Zomer A.L., van Dijk A., Haagsman H.P., Han X. (2018). Immunomodulation and effects on microbiota after in ovo administration of chicken cathelicidin-2. PLoS ONE.

[15] Dunislawska A., Slawinska A., Bednarczyk M., Siwek M. (2019). Transcriptome modulation by in ovo delivered Lactobacillus synbiotics in a range of chicken tissues. Gene.

[16] Siwek M., Slawinska A., Stadnicka K., Bogucka J., Dunislawska A., Bednarczyk M. (2018). Prebiotics and synbiotics—In ovo delivery for improved lifespan condition in chicken. BMC Vet. Res..

[17] Caporaso J.G., Kuczynski J., Stombaugh J., Bittinger K., Bushman F.D., Costello E.K., Fierer N., Pena A.G., Goodrich J.K., Gordon J.I. (2010). QIIME allows analysis of high-throughput community sequencing data. Nat. Methods..

[18] Chen H., Jiang W. (2014). Application of high-throughput sequencing in understanding human oral microbiome related with health and disease. Front. Microbiol..

[19] Gill S.R., Pop M., Deboy R.T., Eckburg P.B., Turnbaugh P.J., Samuel B.S., Gordon J.I., Relman D.A., Fraser-Liggett C.M., Nelson K.E. (2006). Metagenomic analysis of the human distal gut microbiome. Science.

[20] Magoc T., Salzberg S.L. (2011). FLASH: Fast length adjustment of short reads to improve genome assemblies. Bioinformatics.

[21] Edgar R.C. (2010). Search and clustering orders of magnitude faster than BLAST. Bioinformatics.

[22] Desantis T.Z., Hugenholtz P., Larsen N., Rojas M., Brodie E.L., Keller K., Huber T., Dalevi D., Hu P., Andersen G.L. (2006). Greengenes, a chimera-checked 16S rRNA gene database and workbench compatible with ARB. Appl. Environ. Microbiol..

[23] Altschul S.F., Madden T.L., Schaffer A.A., Zhang J., Zhang Z., Miller W., Lipman D.J. (1997). Gapped BLAST and PSI-BLAST: A new generation of protein database search programs. Nucleic Acids Res..

[24] Ramette A. (2007). Multivariate analyses in microbial ecology. FEMS Microbiol. Ecol..

[25] Brian H.M., Marti J.A. (2001). Fitting multivariate models to community data: A comment on distance-based redundancy analysis. Ecology.

[26] Clarke K.R. (1993). Non-parametric multivariate analyses of changes in community structure. Aust. J. Ecol..

[27] Warton D.I., Wright S.T., Wang Y. (2012). Distance-based multivariate analyses confound location and dispersion effects. Methods Ecol. Evol..

[28] Huson D.H., Mitra S., Ruscheweyh H.J., Weber N., Schuster S.C. (2011). Integrative analysis of environmental sequences using MEGAN4. Genome Res..

[29] Asnicar F., Weingart G., Tickle T.L., Huttenhower C., Segata N. (2015). Compact graphical representation of phylogenetic data and metadata with GraPhlAn. PeerJ.

[30] Zaura E., Keijser B.J., Huse S.M., Crielaard W. (2009). Defining the healthy “core microbiome” of oral microbial communities. BMC Microbiol..

[31] White J.R., Nagarajan N., Pop M. (2009). Statistical methods for detecting differentially abundant features in clinical metagenomic samples. PLoS Comput. Biol..

[32] Segata N., Izard J., Waldron L., Gevers D., Miropolsky L., Garrett W.S., Huttenhower C. (2011). Metagenomic biomarker discovery and explanation. Genome Biol..

[33] Chen Y., Yang F., Lu H., Wang B., Chen Y., Lei D., Wang Y., Zhu B., Li L. (2011). Characterization of fecal microbial communities in patients with liver cirrhosis. Hepatology.

[34] Langille M.G., Zaneveld J., Caporaso J.G., Mcdonald D., Knights D., Reyes J.A., Clemente J.C., Burkepile D.E., Vega T.R., Knight R. (2013). Predictive functional profiling of microbial communities using 16S rRNA marker gene sequences. Nat. Biotechnol..

[35] Dominguez-Bello M.G., De Jesus-Laboy K.M., Shen N., Cox L.M., Amir A., Gonzalez A., Bokulich N.A., Song S.J., Hoashi M., Rivera-Vinas J.I. (2016). Partial restoration of the microbiota of cesarean-born infants via vaginal microbial transfer. Nat. Med..

[36] Knights D., Kuczynski J., Charlson E.S., Zaneveld J., Mozer M.C., Collman R.G., Bushman F.D., Knight R., Kelley S.T. (2011). Bayesian community-wide culture-independent microbial source tracking. Nat. Methods.

[37] Ding J., Dai R., Yang L., He C., Xu K., Liu S., Zhao W., Xiao L., Luo L., Zhang Y. (2017). Inheritance and Establishment of Gut Microbiota in Chickens. Front. Microbiol..

[38] Liu H., Ding P., Tong Y., He X., Yin Y., Zhang H., Song Z. (2021). Metabolomic analysis of the egg yolk during the embryonic development of broilers. Poult. Sci..

[39] Damms-Machado A., Mitra S., Schollenberger A.E., Kramer K.M., Meile T., Konigsrainer A., Huson D.H., Bischoff S.C. (2015). Effects of surgical and dietary weight loss therapy for obesity on gut microbiota composition and nutrient absorption. BioMed Res. Int..

[40] Gao P., Liu Y., Le B., Qin B., Liu M., Zhao Y., Guo X., Cao G., Liu J., Li B. (2019). A comparison of dynamic distributions of intestinal microbiota between Large White and Chinese Shanxi Black pigs. Arch. Microbiol..

[41] Indiani C., Rizzardi K.F., Castelo P.M., Ferraz L., Darrieux M., Parisotto T.M. (2018). Childhood Obesity and Firmicutes/Bacteroidetes Ratio in the Gut Microbiota: A Systematic Review. Child Obes..

[42] Yadgary L., Kedar O., Adepeju O., Uni Z. (2013). Changes in yolk sac membrane absorptive area and fat digestion during chick embryonic development. Poult. Sci..

[43] de Faria Ghetti F., Oliveira D.G., de Oliveira J.M., de Castro Ferreira L.E.V.V., Cesar D.E., Moreira A.P.B. (2018). Influence of gut microbiota on the development and progression of nonalcoholic steatohepatitis. Eur. J. Nutr..

[44] Zhang-Sun W., Terce F., Burcelin R., Novikov A., Serino M., Caroff M. (2019). Structure function relationships in three lipids A from the Ralstonia genus rising in obese patients. Biochimie.

[45] Cogburn L.A., Trakooljul N., Chen C., Huang H., Wu C.H., Carre W., Wang X., White H.R. (2018). Transcriptional profiling of liver during the critical embryo-to-hatchling transition period in the chicken (Gallus gallus). BMC Genom..

[46] Xu E., Zhang L., Yang H., Shen L., Feng Y., Ren M., Xiao Y. (2019). Transcriptome profiling of the liver among the prenatal and postnatal stages in chickens. Poult. Sci..

[47] Zhang H., Li H., Kidrick J., Wong E.A. (2019). Localization of cells expressing SGLT1 mRNA in the yolk sac and small intestine of broilers. Poult. Sci..

[48] Uni Z., Geyra A., Ben-Hur H., Sklan D. (2000). Small intestinal development in the young chick: Crypt formation and enterocyte proliferation and migration. Brit. Poult. Sci..

[49] Uni Z., Tako E., Gal-Garber O., Sklan D. (2003). Morphological, molecular, and functional changes in the chicken small intestine of the late-term embryo. Poult. Sci..

[50] Alessi A.M., Gray V., Farquharson F.M., Flores-Lopez A., Shaw S., Stead D., Wegmann U., Shearman C., Gasson M., Collie-Duguid E. (2020). beta-Glucan is a major growth substrate for human gut bacteria related to Coprococcus eutactus. Environ. Microbiol..

[51] Kim J.S., Park J.E., Lee K.C., Choi S.H., Oh B.S., Yu S.Y., Eom M.K., Kang S.W., Han K.I., Suh M.K. (2020). Blautia faecicola sp. nov., isolated from faeces from a healthy human. Int. J. Syst. Evol. Microbiol..

[52] Del C.F., Nobili V., Vernocchi P., Russo A., De Stefanis C., Gnani D., Furlanello C., Zandona A., Paci P., Capuani G. (2017). Gut microbiota profiling of pediatric nonalcoholic fatty liver disease and obese patients unveiled by an integrated meta-omics-based approach. Hepatology.

[53] Ferreira-Halder C.V., Faria A., Andrade S.S. (2017). Action and function of Faecalibacterium prausnitzii in health and disease. Best Pract. Res. Clin. Gastroenterol..

[54] Tamanai-Shacoori Z., Smida I., Bousarghin L., Loreal O., Meuric V., Fong S.B., Bonnaure-Mallet M., Jolivet-Gougeon A. (2017). Roseburia spp.: A marker of health?. Future Microbiol..

